# Surviving or Thriving? Tenets of Shared Decision-Making in Communicating Perioperative Risk Prior to High-Risk Cardiac Surgery

**DOI:** 10.1093/icvts/ivag234

**Published:** 2026-09-04

**Authors:** Ali Hage, Fadi Hage, Caroline A Magro, Aubrey Galloway

**Affiliations:** Division of Cardiovascular Surgery, Peter Munk Cardiac Centre, Toronto General Hospital, University of Toronto, Toronto, ON M5G 2N2, Canada; Department of Cardiothoracic Surgery, Lankenau Heart Institute, Main Line Health, Wynnewood, PA 19096, United States; Department of Cardiothoracic Surgery, NYU Langone Health, New York, NY 10016, United States; Department of Cardiothoracic Surgery, NYU Langone Health, New York, NY 10016, United States

“First, do no harm” should guide decision-making in managing complex patients with indications for cardiac surgery. Today, advances in surgical techniques and technologies allow us to operate on patients who would have previously been deemed inoperable,^1^^,^^2^ including those with advanced heart failure (HF) and high perioperative risk, as quantified by risk calculators (eg, Society of Thoracic Surgeons (STS), and the European System for Cardiac Operative Risk Evaluation (EuroSCORE)) (**Table 1**).^3^^,^^4^ We seek to illustrate tenets that are essential in preoperative conversations with high-risk patients, highlighting the importance of shared decision-making in situations in which, even though we *can* operate, we maybe should not.

**Table 1.** Commonly Used Risk Scores in Cardiac Surgery

Score	Key inputs	Outputs
STS risk score	Age Sex BMI Cardiac status *LVEF, NYHA class, recent MI* Comorbidities *DM, HTN, COPD, PAD, CVD* Renal function Functional status Preoperative status and urgency Reoperation status Procedure	The STS risk score outputs a predicted probability of mortality or major morbidity.
EuroSCORE II	Age Sex Cardiac status *LVEF, NYHA class, recent MI, PHTN* Comorbidities *DM, COPD, extracardiac arteriopathy* Renal function Functional status *Mobility* Preoperative status and urgency Reoperation status Procedure	The EuroSCORE II outputs a predicted probability of in-hospital or 30-day mortality.
TRISCORE	Age Cardiac status *LVEF*, *NYHA class, right-sided HF* Renal function Diuretic use Bilirubin	The TRISCORE outputs a predicted probability of in-hospital mortality after isolated tricuspid valve surgery.
ACEF score	Age Cardiac status *LVEF* Renal function *Serum creatinine*	The ACEF score outputs a simple operative mortality risk, intended for use as a bedside estimate.
SYNTAX II score	Age Sex Cardiac status *LVEF* Comorbidities *COPD, PAD* Renal function Coronary anatomy	The SYNTAX II score outputs a 4-year predicted mortality for PCI versus CABG.

Abbreviations: ACEF = Age-Creatinine-Ejection Fraction; BMI = body mass index; CABG = coronary artery bypass grafting; COPD = chronic obstructive pulmonary disease; CVD = cerebrovascular disease; DM = diabetes mellitus; HF = heart failure; HTN = hypertension; LVEF = left ventricular ejection fraction; MI = myocardial infarction; NYHA = New York Heart Association; PAD = peripheral arterial disease; PCI = percutaneous coronary intervention; PHTN = pulmonary hypertension; STS = Society of Thoracic Surgeons.

Here, to describe the clinical implications of this concept, we present a 78-year-old patient with a past medical history of insulin-dependent diabetes, chronic kidney disease, chronic obstructive pulmonary disease, and coronary artery disease with previous coronary artery bypass grafting. He initially presented to his primary care physician (PCP) with dyspnoea on exertion and fatigue that had limited his functional capacity for multiple months. Echocardiography revealed severe aortic valve stenosis, and his symptoms were determined to be consistent with New York Heart Association Class III HF. He was discussed by the multidisciplinary heart team,^5^ and his perioperative risk was calculated (STS: 18%; EuroSCORE: 15%).^3^^,^^4^ Ultimately, this risk was thought to prohibit a surgical approach, and transcatheter aortic valve replacement (TAVR) was recommended.^6^

He returned to his cardiologist 6 months post-TAVR, endorsing dyspnoea and further reductions in functional capacity. Echocardiography revealed paravalvular aortic regurgitation with a reduction in left ventricular ejection fraction. He was re-discussed by the heart team,^5^ many of whom were challenged by questions such as, “What are we losing if we operate? We *can* perform a surgical aortic valve replacement (SAVR), and it is the last chance this patient has to survive.” He was therefore referred to our surgical clinic for evaluation for SAVR, despite previous denial due to perioperative risk.

Now, the surgical team is tasked with shepherding this high-risk patient towards the best next step in his cardiac care. It is attractive to rely on risk scores (eg, STS, EuroSCORE)^3^^,^^4^ to communicate perioperative risk in such scenarios. However, “50% risk of morbidity and mortality,” as determined by a risk calculator alone, may optimistically be interpreted as a “50% chance to survive and thrive,” akin to winning the lottery after years of functional decline. It is our responsibility to ensure that risk is adequately understood, and we offer a framework to communicate the potentially complex consequences of cardiac surgery. We suggest that this approach be adopted by surgeons, cardiologists, and PCPs to streamline conversations throughout the continuum of cardiac care.

1. ***Discussion of Short-Term Risks of Surgery***: We must address the short-term risks of surgery, including bleeding, renal failure, respiratory complications, cerebrovascular complications, and the need for pharmacological and mechanical circulatory support. It is important to paint a realistic picture of the perioperative period, including the possibility of prolonged admission to the intensive care unit (ICU).^7^ Undoubtedly, it is difficult for families to encounter a loved one on a ventilator, extracorporeal membrane oxygenation, or continuous renal replacement therapy,^8^ and we must introduce the potential need for such support prior to the operating room (OR).
2. ***Discussion of Long-Term Risks of Surgery***: Additionally, mid- and long-term risks must be described, as risk calculators often fail to capture the impact of high-risk surgery on quality of life beyond the perioperative period. It is common for patients to anticipate a prompt discharge home and return to “normalcy,” and we must prepare them for the possibility of subacute rehabilitation or a long-term care facility. Further, a number of short-term risks (eg, renal failure, respiratory complications, and cerebrovascular complications), as previously described, are associated with sequelae that may impact quality of life for years after the index admission.
3. ***Anticipated Outcomes***: We are often asked to prognosticate the course of our patients despite limited information and a vast spectrum of potential outcomes. A risk score offers insights into the likelihood of survival, morbidity, and mortality, though clinical acumen is necessary in counselling our patients. We suggest the discussion of best-case and worst-case scenarios to facilitate decision-making in the context of uncertainty. In speaking with our high-risk patient, for example, we would highlight symptomatic relief and recovery of cardiac function as best-case scenarios; worst-case scenarios would involve intraoperative and perioperative complications, with the potential for prolonged admission and significant morbidity and mortality.
4. ***Alternatives to Surgery***: We consider the frequently encountered scenario in which the family of our high-risk patient asks, “What would you do if this was your father (brother, husband, etc.)? Would you not offer him the surgery?” We must respond to such queries with both empathy and evidence to inform an individualized approach for our patient. We underscore the importance of multidisciplinary discussion with cardiologists and cardiac surgeons, such that patients are aware of all medical, interventional, and surgical strategies available to them.
5. ***Goals of Care***: Lastly, we should strive to understand the unique values of our patients to prioritize their wishes within each step of their cardiac care. We emphasize the importance of transparent conversations regarding goals of care (eg, longevity, independence, freedom from hospitalization). Clearly, shared decision-making will differ when speaking with a patient who wishes to pursue all available interventions, as compared to one with no interest in surgery.^9^^,^^10^

Daily, our patients enter our clinics and entrust us with their hearts, appreciating our honest thoughts and opinions. Our approach to high-risk cases cannot be paternalistic, but neither should the entire weight of difficult decisions be placed on patients and their families. We believe that the best approach centres upon a discussion of the myriad of potential perioperative outcomes, followed by shared decision-making. Physicians and surgeons should not simply parrot risk scores, but rather communicate the likelihood of best-case and worst-case scenarios, as informed by individual goals of care. We cannot be too busy to lead these discussions, which justify the trust we receive from our patients within and beyond the OR.

## Data Availability

No data were generated nor analyzed for the purposes of this editorial; there are no data to make available.
